# Bis(*N*,*N*,*N*-trimethyl­ethanaminium) bis­(1,4-tetra­selenido-κ^2^ *Se* ^1^,*Se* ^4^)cadmate

**DOI:** 10.1107/S1600536811007227

**Published:** 2011-03-02

**Authors:** Jaemyeong Kim, Kang-Woo Kim

**Affiliations:** a Department of Chemistry, University of Incheon, Incheon 406-772, Republic of Korea

## Abstract

The title compound, (EtMe_3_N)_2_[Cd(Se_4_)_2_], which has been prepared by reaction of CdI_2_, K_2_Se_4_ and EtMe_3_NI in dimethyl­formamide, is the first example of a [Cd(Se_4_)_2_]^2−^ anion stabilized by alkyl­ammonium counter-ions. The Cd atom in the complex [Cd(Se_4_)_2_]^2−^ anion is tetra­hedrally coordinated by two chelating tetra­selenide ligands, and both CdSe_4_ rings exhibit an envelope conformation.

## Related literature

For general background to [Cd(Se_4_)_2_]^2−^ complexes, see: Kanatzidis & Huang (1994 ▶); Ansari *et al.* (1990 ▶); Barrie *et al.* (1994 ▶). For related structures, see: Adel *et al.* (1988 ▶); Kräuter *et al.* (1989 ▶); Magull *et al.* (1992 ▶); Banda *et al.* (1989 ▶). For applications of soluble cadmium–chalcogen compounds, see: Khanna *et al.* (2006) ▶; Nesheva (2001 ▶); Dhingra *et al.* (1991 ▶).
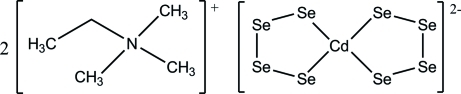


## Experimental

### 

#### Crystal data

(C_5_H_14_N)_2_[Cd(Se_4_)_2_]
*M* *_r_* = 920.42
Monoclinic, 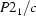

*a* = 12.5125 (2) Å
*b* = 11.3273 (2) Å
*c* = 16.7290 (3) Å
β = 95.174 (1)°
*V* = 2361.39 (7) Å^3^
*Z* = 4
Mo *K*α radiation
μ = 13.25 mm^−1^
*T* = 173 K
0.30 × 0.27 × 0.16 mm

#### Data collection

Bruker APEXII CCD ULTRA diffractometer
Absorption correction: multi-scan (*SADABS*; Bruker, 2005 ▶) *T*_min_ = 0.010, *T*_max_ = 0.041
40764 measured reflections
5876 independent reflections
4911 reflections with *I* > 2σ(*I*)
*R*_int_ = 0.074

#### Refinement

*R*[*F* ^2^ > 2σ(*F* ^2^)] = 0.032
*wR*(*F* ^2^) = 0.081
*S* = 1.03
5876 reflections
191 parameters
H-atom parameters constrained
Δρ_max_ = 0.88 e Å^−3^
Δρ_min_ = −1.85 e Å^−3^

### 

Data collection: *APEX2* (Bruker, 2005 ▶); cell refinement: *SAINT* (Bruker, 2005 ▶); data reduction: *SAINT*; program(s) used to solve structure: *SHELXS97* (Sheldrick, 2008 ▶); program(s) used to refine structure: *SHELXL97* (Sheldrick, 2008 ▶); molecular graphics: *DIAMOND* (Brandenburg, 2006 ▶); software used to prepare material for publication: *WinGX* (Farrugia, 1999 ▶).

## Supplementary Material

Crystal structure: contains datablocks global, I. DOI: 10.1107/S1600536811007227/zl2349sup1.cif

Structure factors: contains datablocks I. DOI: 10.1107/S1600536811007227/zl2349Isup2.hkl

Additional supplementary materials:  crystallographic information; 3D view; checkCIF report

## Figures and Tables

**Fig. 1.**
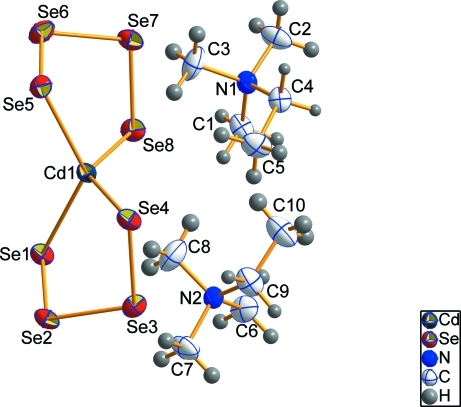
Structure of the asymmetric unit in (EtMe3N)2[Cd(Se4)2]. Displacement ellipsoids are drawn at the 50% probability level, except for H atoms which are drawn as spheres with an arbitrary radius.

## supplementary crystallographic information

### Comment

Within metal polychalcogenide chemistry, the bis(tetrachalcogenido)metallate [*M*(Q_4_)_2_]^2-^ (Q = S, Se, Te) anions are among the most well known molecular complexes (Kanatzidis & Huang (1994); Ansari *et al.* (1990)). For the [Cd(Se_4_)_2_]^2-^ anion, there are four structurally characterized complexes: stabilized with [Na(15-crown-5]^+^ (Adel *et al.* (1988)), [Li_3_(12-crown-4)_3_(CH_3_COO]^2+^ (Kräuter *et al.* (1989)), [Ba(18-crown-6)(DMF)_4_]^2+^ (Magull *et al.* (1992)), and [Ph_4_P]^+^ (Banda *et al.* (1989)). So far, no alkylammonium salt of the [Cd(Se_4_)_2_]^2-^ anion had been structurally characterized. Compared to alkali metal-crown ether complexes and arylphosphonium salts, an alkylammonium salt could be preferable for the application as a precursor of Cd/Se binary and related materials (Khanna *et al.* (2006); Nesheva (2001); Dhingra *et al.* (1991)), and also for ^77^Se solid-state NMR measurements (Barrie *et al.* (1994)). The title compound is the first example of an alkylammonium [Cd(Se_4_)_2_]^2-^ salt, containing EtMe_3_N^+^ cations as the counterion.

The structure of the [Cd(Se_4_)_2_]^2-^ anion in (EtMe_3_N)_2_[Cd(Se_4_)_2_] is essentially the same as that of the [Na(15-crown-5]^+^, [Li_3_(12-crown-4)_3_(CH_3_COO]^2+^, [Ba(18-crown-6)(DMF)_4_]^2+^, and [Ph_4_P]^+^ salts. As shown in Fig. 1, a Cd atom is tetrahedrally coordinated by two chelating tetraselenide ligands, and all eight Se atoms occupy distinct crystallographic sites. The Cd—Se distances and Se—Se distances are typical, ranging from 2.6347 (5) Å to 2.6789 (5) Å, and from 2.3289 (6) Å to 2.3419 (6) Å, respectively, similar to those found in the previously characterized four [Cd(Se_4_)_2_]^2-^ complexes. Both CdSe_4_ rings in [Cd(Se_4_)_2_]^2-^ exhibit the envelope conformation. In one CdSe_4_ ring, the Cd1, Se1, Se2, and Se4 atoms are considered to be in a plane with a mean deviation of 0.12 (4) Å, while the Se3 atom lies 1.24 Å below it. Similarly, in the other CdSe_4_ ring, the Cd1, Se5, Se7, and Se8 atoms are in a plane with a mean deviation of 0.15 (6) Å, and the Se6 atom lies 1.17 Å below it. The dihedral angle between the two planes in [Cd(Se_4_)_2_]^2-^ is found to be 112.12°.

### Experimental

All synthetic experiments were performed under an atmosphere of dry argon or nitrogen using either a glove box or a Schlenk line. To a 50 ml DMF solution of 0.59 g (1.5 mmol) K_2_Se_4_ and 0.32 g (1.5 mmol) EtMe_3_NI, a 10 ml DMF solution of 0.27 g (0.75 mmol) CdI_2_ was added dropwise over a 20 min period. 60 ml ether were slowly layered over the filtrate solution, after removing undissolved precipitates by filtration. Upon standing at room temperature for 3 days, dark purple crystals were obtained. These crystals were isolated and washed with ether several times. More crystals were obtained upon layering additional 50 ml ether over the solution after isolation of the first crop of crystals. The overall yield was 65%, based on the CdI_2_ used. SEM/EDAX analyses on the crystals of (EtMe_3_N)_2_[Cd(Se_4_)_2_] showed an average Cd:Se atomic ratio of 1:7.2.

### Refinement

H atoms were positioned geometrically and treated as riding, with C—H = 0.97 (CH_2_) and 0.96 (CH_3_) Å with *U*_iso_(H) = 1.2 (1.5 for methyl) *U*_eq_(C). H atoms of the CH_3_ were positioned to be staggered with respect to the shortest other bond to the atom to which the CH_3_ is attached.

### Crystal data

**Table:**

(C_5_H_14_N)_2_[Cd(Se_4_)_2_]	*F*(000) = 1688
*M**_r_* = 920.42	*D*_x_ = 2.589 Mg m^−^^3^
Monoclinic, *P*2_1_/*c*	Mo *K*α radiation, λ = 0.71073 Å
*a* = 12.5125 (2) Å	Cell parameters from 9912 reflections
*b* = 11.3273 (2) Å	θ = 2.4–28.1°
*c* = 16.7290 (3) Å	µ = 13.25 mm^−^^1^
β = 95.174 (1)°	*T* = 173 K
*V* = 2361.39 (7) Å^3^	Polyhedral block, dark purple
*Z* = 4	0.30 × 0.27 × 0.16 mm

### Data collection

**Table:**

Bruker APEXII CCD ULTRA diffractometer	5876 independent reflections
Radiation source: Turbo X-ray	4911 reflections with *I* > 2σ(*I*)
Multilyar	*R*_int_ = 0.074
φ and ω scans	θ_max_ = 28.3°, θ_min_ = 1.6°
Absorption correction: multi-scan (*SADABS*; Bruker, 2005)	*h* = −16→16
*T*_min_ = 0.010, *T*_max_ = 0.041	*k* = −15→15
40764 measured reflections	*l* = −22→20

### Refinement

**Table:**

Refinement on *F*^2^	Secondary atom site location: difference Fourier map
Least-squares matrix: full	Hydrogen site location: inferred from neighbouring sites
*R*[*F*^2^ > 2σ(*F*^2^)] = 0.032	H-atom parameters constrained
*wR*(*F*^2^) = 0.081	*w* = 1/[σ^2^(*F*_o_^2^) + (0.0326*P*)^2^ + 1.8487*P*] where *P* = (*F*_o_^2^ + 2*F*_c_^2^)/3
*S* = 1.03	(Δ/σ)_max_ = 0.001
5876 reflections	Δρ_max_ = 0.88 e Å^−^^3^
191 parameters	Δρ_min_ = −1.85 e Å^−^^3^
0 restraints	Extinction correction: *SHELXL97* (Sheldrick, 2008)
Primary atom site location: structure-invariant direct methods	Extinction coefficient: 0.00141 (10)

### Special details

**Table:**

Geometry. All s.u.'s (except the s.u. in the dihedral angle between two l.s. planes) are estimated using the full covariance matrix. The cell s.u.'s are taken into account individually in the estimation of s.u.'s in distances, angles and torsion angles; correlations between s.u.'s in cell parameters are only used when they are defined by crystal symmetry. An approximate (isotropic) treatment of cell s.u.'s is used for estimating s.u.'s involving l.s. planes.
Refinement. Refinement of *F*^2^ against ALL reflections. The weighted *R*-factor *wR* and goodness of fit *S* are based on *F*^2^, conventional *R*-factors *R* are based on *F*, with *F* set to zero for negative *F*^2^. The threshold expression of *F*^2^ > 2σ(*F*^2^) is used only for calculating *R*-factors(gt) *etc*. and is not relevant to the choice of reflections for refinement. *R*-factors based on *F*^2^ are statistically about twice as large as those based on *F*, and *R*- factors based on ALL data will be even larger.

### Fractional atomic coordinates and isotropic or equivalent isotropic displacement parameters (Å^2^)

**Table:**

	*x*	*y*	*z*	*U*_iso_*/*U*_eq_	
Cd1	0.28864 (2)	0.52306 (3)	0.255978 (17)	0.02603 (9)	
Se1	0.29708 (3)	0.75340 (4)	0.28334 (3)	0.03026 (11)	
Se2	0.47862 (3)	0.76914 (4)	0.32974 (3)	0.03032 (11)	
Se3	0.49898 (3)	0.60822 (4)	0.41770 (3)	0.03229 (11)	
Se4	0.47131 (3)	0.44849 (4)	0.32945 (3)	0.03102 (11)	
Se5	0.28100 (3)	0.40649 (4)	0.11800 (2)	0.03112 (11)	
Se6	0.09505 (3)	0.40696 (5)	0.08845 (3)	0.03800 (12)	
Se7	0.03406 (3)	0.34066 (4)	0.20879 (3)	0.03345 (11)	
Se8	0.08684 (3)	0.49621 (4)	0.29446 (3)	0.03304 (11)	
N1	0.2702 (3)	0.1209 (3)	0.3584 (2)	0.0271 (7)	
N2	0.1899 (2)	0.6780 (3)	0.5421 (2)	0.0273 (7)	
C1	0.2527 (4)	0.2362 (4)	0.3994 (3)	0.0363 (10)	
H1A	0.2383	0.2217	0.4539	0.054*	
H1B	0.1929	0.2766	0.3718	0.054*	
H1C	0.3159	0.2842	0.3988	0.054*	
C2	0.1710 (4)	0.0470 (5)	0.3612 (3)	0.0451 (12)	
H2A	0.1575	0.034	0.4161	0.068*	
H2B	0.1811	−0.0275	0.3356	0.068*	
H2C	0.111	0.0873	0.3337	0.068*	
C3	0.2907 (4)	0.1413 (5)	0.2737 (3)	0.0500 (14)	
H3A	0.354	0.1888	0.2718	0.075*	
H3B	0.2305	0.1815	0.2463	0.075*	
H3C	0.301	0.0669	0.248	0.075*	
C4	0.3621 (3)	0.0540 (4)	0.4028 (3)	0.0316 (9)	
H4A	0.3443	0.0394	0.4572	0.038*	
H4B	0.3687	−0.022	0.377	0.038*	
C5	0.4685 (4)	0.1152 (5)	0.4065 (3)	0.0462 (12)	
H5A	0.5223	0.0659	0.4341	0.069*	
H5B	0.4643	0.1886	0.4347	0.069*	
H5C	0.4871	0.1302	0.353	0.069*	
C6	0.1112 (4)	0.7104 (5)	0.5999 (3)	0.0426 (11)	
H6A	0.1461	0.7101	0.6534	0.064*	
H6B	0.0535	0.6542	0.5963	0.064*	
H6C	0.0832	0.7877	0.5874	0.064*	
C7	0.2779 (4)	0.7675 (5)	0.5477 (3)	0.0489 (13)	
H7A	0.3118	0.7692	0.6015	0.073*	
H7B	0.2486	0.8439	0.534	0.073*	
H7C	0.3299	0.7469	0.5112	0.073*	
C8	0.1357 (4)	0.6786 (6)	0.4594 (3)	0.0561 (15)	
H8A	0.0804	0.6196	0.4549	0.084*	
H8B	0.1873	0.6618	0.4217	0.084*	
H8C	0.1045	0.7549	0.4479	0.084*	
C9	0.2423 (4)	0.5611 (5)	0.5627 (3)	0.0434 (12)	
H9A	0.2962	0.5465	0.5257	0.052*	
H9B	0.2791	0.5665	0.6162	0.052*	
C10	0.1678 (5)	0.4572 (5)	0.5604 (4)	0.0588 (15)	
H10A	0.2083	0.3867	0.5732	0.088*	
H10B	0.1312	0.4502	0.5077	0.088*	
H10C	0.1162	0.4684	0.5989	0.088*	

### Atomic displacement parameters (Å^2^)

**Table:**

	*U*^11^	*U*^22^	*U*^33^	*U*^12^	*U*^13^	*U*^23^
Cd1	0.02102 (14)	0.02943 (17)	0.02811 (16)	−0.00364 (11)	0.00481 (11)	0.00007 (11)
Se1	0.02386 (19)	0.0284 (2)	0.0385 (2)	0.00127 (16)	0.00277 (16)	0.00038 (17)
Se2	0.02108 (19)	0.0273 (2)	0.0435 (3)	−0.00169 (16)	0.00789 (16)	−0.00507 (17)
Se3	0.0269 (2)	0.0401 (3)	0.0298 (2)	0.00456 (18)	0.00195 (16)	−0.00306 (17)
Se4	0.02296 (19)	0.0274 (2)	0.0426 (3)	0.00099 (16)	0.00250 (17)	0.00039 (17)
Se5	0.0253 (2)	0.0401 (3)	0.0292 (2)	−0.00149 (17)	0.00922 (16)	−0.00414 (17)
Se6	0.0279 (2)	0.0541 (3)	0.0313 (2)	0.0017 (2)	−0.00101 (17)	−0.00473 (19)
Se7	0.01889 (19)	0.0374 (3)	0.0446 (3)	−0.00443 (17)	0.00541 (16)	−0.00307 (19)
Se8	0.0223 (2)	0.0426 (3)	0.0357 (2)	−0.00182 (17)	0.01091 (16)	−0.00686 (18)
N1	0.0189 (15)	0.0344 (19)	0.0293 (18)	0.0003 (14)	0.0093 (13)	−0.0002 (14)
N2	0.0223 (15)	0.0346 (19)	0.0260 (17)	−0.0016 (14)	0.0074 (12)	−0.0010 (14)
C1	0.033 (2)	0.029 (2)	0.049 (3)	0.0038 (18)	0.0139 (19)	0.0045 (19)
C2	0.025 (2)	0.044 (3)	0.066 (3)	−0.007 (2)	0.009 (2)	−0.006 (2)
C3	0.043 (3)	0.080 (4)	0.029 (3)	0.013 (3)	0.011 (2)	0.011 (2)
C4	0.028 (2)	0.032 (2)	0.035 (2)	0.0071 (18)	0.0075 (17)	0.0023 (17)
C5	0.025 (2)	0.051 (3)	0.062 (3)	0.000 (2)	−0.002 (2)	0.001 (2)
C6	0.032 (2)	0.058 (3)	0.040 (3)	−0.002 (2)	0.0186 (19)	−0.011 (2)
C7	0.049 (3)	0.047 (3)	0.054 (3)	−0.019 (2)	0.024 (2)	−0.010 (2)
C8	0.048 (3)	0.088 (5)	0.032 (3)	0.007 (3)	−0.002 (2)	0.001 (3)
C9	0.029 (2)	0.042 (3)	0.060 (3)	0.004 (2)	0.006 (2)	0.008 (2)
C10	0.055 (3)	0.043 (3)	0.081 (4)	−0.007 (3)	0.022 (3)	0.002 (3)

### Geometric parameters (Å, °)

**Table:**

Cd1—Se4	2.6347 (5)	C3—H3A	0.96
Cd1—Se1	2.6494 (5)	C3—H3B	0.96
Cd1—Se5	2.6535 (5)	C3—H3C	0.96
Cd1—Se8	2.6789 (5)	C4—C5	1.497 (6)
Se1—Se2	2.3402 (5)	C4—H4A	0.97
Se2—Se3	2.3419 (6)	C4—H4B	0.97
Se3—Se4	2.3411 (6)	C5—H5A	0.96
Se5—Se6	2.3346 (6)	C5—H5B	0.96
Se6—Se7	2.3401 (7)	C5—H5C	0.96
Se7—Se8	2.3289 (6)	C6—H6A	0.96
N1—C3	1.481 (6)	C6—H6B	0.96
N1—C1	1.500 (6)	C6—H6C	0.96
N1—C2	1.501 (6)	C7—H7A	0.96
N1—C4	1.515 (5)	C7—H7B	0.96
N2—C8	1.485 (6)	C7—H7C	0.96
N2—C6	1.487 (5)	C8—H8A	0.96
N2—C7	1.494 (6)	C8—H8B	0.96
N2—C9	1.504 (6)	C8—H8C	0.96
C1—H1A	0.96	C9—C10	1.499 (7)
C1—H1B	0.96	C9—H9A	0.97
C1—H1C	0.96	C9—H9B	0.97
C2—H2A	0.96	C10—H10A	0.96
C2—H2B	0.96	C10—H10B	0.96
C2—H2C	0.96	C10—H10C	0.96
			
Se4—Cd1—Se1	102.462 (15)	H3B—C3—H3C	109.5
Se4—Cd1—Se5	102.001 (17)	C5—C4—N1	114.9 (4)
Se1—Cd1—Se5	129.652 (18)	C5—C4—H4A	108.5
Se4—Cd1—Se8	130.416 (18)	N1—C4—H4A	108.5
Se1—Cd1—Se8	95.396 (16)	C5—C4—H4B	108.5
Se5—Cd1—Se8	101.053 (16)	N1—C4—H4B	108.5
Se2—Se1—Cd1	98.899 (19)	H4A—C4—H4B	107.5
Se1—Se2—Se3	101.33 (2)	C4—C5—H5A	109.5
Se4—Se3—Se2	101.76 (2)	C4—C5—H5B	109.5
Se3—Se4—Cd1	96.841 (19)	H5A—C5—H5B	109.5
Se6—Se5—Cd1	98.053 (19)	C4—C5—H5C	109.5
Se5—Se6—Se7	102.31 (2)	H5A—C5—H5C	109.5
Se8—Se7—Se6	100.97 (2)	H5B—C5—H5C	109.5
Se7—Se8—Cd1	99.156 (19)	N2—C6—H6A	109.5
C3—N1—C1	110.3 (4)	N2—C6—H6B	109.5
C3—N1—C2	109.4 (4)	H6A—C6—H6B	109.5
C1—N1—C2	108.5 (3)	N2—C6—H6C	109.5
C3—N1—C4	111.0 (3)	H6A—C6—H6C	109.5
C1—N1—C4	110.3 (3)	H6B—C6—H6C	109.5
C2—N1—C4	107.4 (3)	N2—C7—H7A	109.5
C8—N2—C6	109.4 (3)	N2—C7—H7B	109.5
C8—N2—C7	109.1 (4)	H7A—C7—H7B	109.5
C6—N2—C7	108.6 (4)	N2—C7—H7C	109.5
C8—N2—C9	111.8 (4)	H7A—C7—H7C	109.5
C6—N2—C9	111.6 (4)	H7B—C7—H7C	109.5
C7—N2—C9	106.2 (4)	N2—C8—H8A	109.5
N1—C1—H1A	109.5	N2—C8—H8B	109.5
N1—C1—H1B	109.5	H8A—C8—H8B	109.5
H1A—C1—H1B	109.5	N2—C8—H8C	109.5
N1—C1—H1C	109.5	H8A—C8—H8C	109.5
H1A—C1—H1C	109.5	H8B—C8—H8C	109.5
H1B—C1—H1C	109.5	C10—C9—N2	115.3 (4)
N1—C2—H2A	109.5	C10—C9—H9A	108.4
N1—C2—H2B	109.5	N2—C9—H9A	108.4
H2A—C2—H2B	109.5	C10—C9—H9B	108.4
N1—C2—H2C	109.5	N2—C9—H9B	108.4
H2A—C2—H2C	109.5	H9A—C9—H9B	107.5
H2B—C2—H2C	109.5	C9—C10—H10A	109.5
N1—C3—H3A	109.5	C9—C10—H10B	109.5
N1—C3—H3B	109.5	H10A—C10—H10B	109.5
H3A—C3—H3B	109.5	C9—C10—H10C	109.5
N1—C3—H3C	109.5	H10A—C10—H10C	109.5
H3A—C3—H3C	109.5	H10B—C10—H10C	109.5

## References

[bb1] Adel, J., Weller, F. & Dehnicke, K. (1988). *Z. Naturforsch. Teil B*, **43**, 1094–1100.

[bb2] Ansari, M. A., Mahler, C. H., Chorghade, G. S., Lu, Y.-J. & Ibers, J. A. (1990). *Inorg. Chem.* **29**, 3832–3839.

[bb3] Banda, R. M. H., Cusick, J., Scudder, M. L., Craig, D. C. & Dance, I. G. (1989). *Polyhedron*, **8**, 1995–1998.

[bb4] Barrie, P. J., Clark, R. J. H., Withnall, R., Chung, D.-Y., Kim, K.-W. & Kanatzidis, M. G. (1994). *Inorg. Chem.* **33**, 1212–1216.

[bb5] Brandenburg, K. (2006). *DIAMOND* Crystal Impact GbR, Bonn, Germany.

[bb6] Bruker (2005). *APEX2*, *SAINT* and *SADABS* Bruker AXS Inc., Madison, Wisconsin, USA.

[bb7] Dhingra, S., Kim, K.-W. & Kanatzidis, M. G. (1991). *Mater. Res. Soc. Symp. Proc.* **204**, 163–168.

[bb8] Farrugia, L. J. (1999). *J. Appl. Cryst.* **32**, 837–838.

[bb9] Kanatzidis, M. G. & Huang, S.-P. (1994). *Coord. Chem. Rev.* **130**, 509–621.

[bb10] Khanna, P. K., Singh, N., Charan, S., Lonkar, S. P., Reddy, A. S., Patil, Y. & Viswanath, A. K. (2006). *Mater. Chem. Phys.* **97**, 288–294.

[bb11] Kräuter, G., Weller, F. & Dehnicke, K. (1989). *Z. Naturforsch. Teil B*, **44**, 444–454.

[bb12] Magull, S., Dehnicke, K. & Fenske, D. (1992). *Z. Anorg. Allg. Chem.* **608**, 17–22.

[bb13] Nesheva, D. (2001). *Handbook of Surfaces and Interfaces of Materials*, Vol. 3, edited by H. S. Nalwa, pp. 239–279. San Diego: Academic Press.

[bb14] Sheldrick, G. M. (2008). *Acta Cryst.* A**64**, 112–122. 10.1107/S0108767307043930 18156677

